# Renal toxicity of ifosfamide in children with cancer: an exploratory study integrating aldehyde dehydrogenase enzymatic activity data and a wide-array urinary metabolomics approach

**DOI:** 10.1186/s12887-024-04633-1

**Published:** 2024-03-19

**Authors:** Olivia Febvey-Combes, Jérôme Guitton, Perrine Marec-Berard, Cécile Faure-Conter, Ellen Blanc, Sylvie Chabaud, Agnès Conjard-Duplany, Matthias Schell, Laurence Derain Dubourg

**Affiliations:** 1 Centre Léon Bérard, Direction de la Recherche Clinique et de l’Innovation, Lyon, France; 2 Laboratoire de Pharmacologie et Toxicologie, Hospices Civils de Lyon, Hôpital Lyon Sud, Pierre-Bénite, France; 3 Faculté de Pharmacie, Département de toxicologie, Université Claude Bernard Lyon 1, Lyon, France; 4 Institut d’hématologie et d’oncologie pédiatrique – Centre Léon Bérard, Département d’oncologie pédiatrique, Lyon, France; 5 Faculté de Médecine Lyon Est, Physiopathologie et Génétique du Neurone et du Muscle, Université Lyon1, CNRS UMR 5261, INSERM U1315, Lyon, France; 6 Service de Néphrologie, Hospices Civils de Lyon, Hôpital Edouard Herriot, Dialyse, Hypertension et Exploration Fonctionnelle Rénale 5, place d’Arsonval, Lyon cedex 03, 69437 France; 7 Université Lyon 1, CNRS UMR 5305, Lyon, France

**Keywords:** Childhood cancer, Chemotherapy, Nephrotoxicity, Ifosfamide, Aldehyde dehydrogenase

## Abstract

**Background:**

Ifosfamide is a major anti-cancer drug in children with well-known renal toxicity. Understanding the mechanisms underlying this toxicity could help identify children at increased risk of toxicity.

**Methods:**

The IFOS01 study included children undergoing ifosfamide-based chemotherapy for Ewing sarcoma or rhabdomyosarcoma. A fully evaluation of renal function was performed during and after chemotherapy. Proton nuclear magnetic resonance (NMR) and conventional biochemistry were used to detect early signs of ifosfamide-induced tubulopathy. The enzymatic activity of aldehyde dehydrogenase (ALDH) was measured in the peripheral blood lymphocytes as a marker of ifosfamide-derived chloroacetaldehyde detoxification capacity. Plasma and urine concentrations of ifosfamide and dechloroethylated metabolites were quantified.

**Results:**

The 15 participants received a median total ifosfamide dose of 59 g/m^2^ (range: 24–102), given over a median of 7 cycles (range: 4–14). All children had acute proximal tubular toxicity during chemotherapy that was reversible post-cycle, seen with both conventional assays and NMR. After a median follow-up of 31 months, 8/13 children presented overall chronic toxicity among which 7 had decreased glomerular filtration rate. ALDH enzymatic activity showed high inter- and intra-individual variations across cycles, though overall activity looked lower in children who subsequently developed chronic nephrotoxicity. Concentrations of ifosfamide and metabolites were similar in all children.

**Conclusions:**

Acute renal toxicity was frequent during chemotherapy and did not allow identification of children at risk for long-term toxicity. A role of ALDH in late renal dysfunction is possible so further exploration of its enzymatic activity and polymorphism should be encouraged to improve the understanding of ifosfamide-induced nephrotoxicity.

**Supplementary Information:**

The online version contains supplementary material available at 10.1186/s12887-024-04633-1.

## Introduction

Therapeutic advances in pediatric cancers in the past 50 years have resulted in an increased five-year survival rate from 20 to 80% for all pediatric cancers [1]. Increased survival rates, due in part to multimodal risk-directed therapy, have resulted in a growing population of adolescent and adult cancer survivors who are at risk for long-term complications from therapy. The increase in survival thus requires the clinicians to manage both the acute and the delayed therapy-related toxicities.

Acute toxicities related to therapy are well described and are agent specific. Although these toxicities are usually transient, they may result in significant morbidity and delay in chemotherapy administration. Late effects of childhood cancer therapy are toxicities that are absent or subclinical at the completion of therapy but manifest later in life. It is estimated that 60-70% of childhood cancer survivors experience at least one disability related to their cancer, most commonly therapy-induced [2].

Ifosfamide is a major cancer drug generally given in combination therapy in children, and known to be nephrotoxic. Ifosfamide can induce both tubular and glomerular damages, with high cumulative ifosfamide dose increasing this risk [3, 4]. Proximal tubular injury is the most frequent and severe complication, which can present either as an isolated tubular dysfunction or a complete Fanconi syndrome [5]. These renal alterations may induce losses of phosphate, glucose, or amino acids, as well as tubular acidosis. Nephrotoxicity often occurs during treatment but can also develop months or years after treatment completion. Prior or concomitant treatment with cisplatin, radiation therapy or nephronic reduction are other risk factors of long-term nephrotoxicity reported in the literature [3].

The toxicity of ifosfamide may be related to the production of high quantities of chloroacetaldehyde, which is one of the main products of hepatic ifosfamide metabolism [6]. In fact, up to 50% of ifosfamide may undergo dechloroethylation, resulting in the formation of 2- and 3-dechloroethyl-ifosfamide and chloroacetaldehyde [7]. Chloroacetaldehyde has been shown to be toxic on proximal tubular cells in vitro [8, 9]. However, on the contrary, one report suggested that a decrease in dechloroethylation (and potentially in chloroacetaldehyde production) was associated with an increased risk of nephrotoxicity in patients receiving the drug repeatedly [10]. These observations suggest that the mechanisms underlying ifosfamide-induced nephrotoxicity are not completely understood.

The inter-individual variability in the sensitivity to the nephrotoxic effects of ifosfamide may result from differential exposure of the renal cells to chloroacetaldehyde. Hence, it was hypothesized that an increase in chloroacetaldehyde levels may be related to an increased production by the liver and/or the kidney, and/or a reduced catabolic activity of aldehyde dehydrogenase (ALDH) that turns toxic chloroacetaldehyde into inactive chloroacetate [8].

The present study analyzed ALDH enzymatic activity as a marker of chloroacetaldehyde detoxification, and quantified plasma concentration of ifosfamide and its dechloroethylated metabolites as indicators of chloroacetaldehyde production. The relevance of the association of routine biochemical parameters with nuclear magnetic resonance (NMR) spectroscopy for the early detection of subtle, ifosfamide-induced tubular abnormalities was also examined.

## Methods

### Participants

Participants were recruited in the department of pediatric oncology at the Léon Bérard Comprehensive Cancer Center, Lyon, France, from 2009 to 2015. Inclusion criteria were: children aged 6 to 18 years with a first primary Ewing sarcoma or rhabdomyosarcoma scheduled to receive ifosfamide-based chemotherapy regimen, a life expectancy > 8 weeks, no prior radiation or chemotherapy, no history of congenital or acquired kidney or liver disease, no recent history or ongoing nocturnal enuresis, and no liver or renal failure.

### Chemotherapy regimen

Induction chemotherapy consisted of 9 cycles of IVA regimen in patients with rhabdomyosarcoma (ifosfamide 3000 mg/m^2^/day for two consecutive days, and vincristine and actinomycin D at the dose of 1.5 mg/m^2^ on day 1 of each cycle). In patients with Ewing sarcoma, induction treatment included 6 cycles of VIDE regimen (vincristine 1.5 mg/m^2^ on day 1 of each cycle, and ifosfamide 3000 mg/m^2^/day, adriamycine 20 mg/m^2^/day, and etoposide 150 mg/m^2^/day for three consecutive days). In all regimens, ifosfamide was administered intravenously using a central venous line as a 3-hour infusion in combination with uromitexan. All regimens included 3 L/m^2^/day of continuous intravenous hyperhydration (e.g. Glucidion®, or Bionolyte®) started at least 3 h prior to the initiation of ifosfamide. All patients received a 24-hour post-chemotherapy hyperhydration per protocol.

### Plasma and urine samples

At each chemotherapy cycle **(**i.e. 6 cycles of VIDE or 9 cycles of IVA), blood and urine samples were collected in fasting condition.

#### Blood

Blood samples were collected from the central intravenous line, prior to ifosfamide initiation and 24 h after the completion of the last dose of ifosfamide. Peripheral blood lymphocytes were isolated from patients’ blood sample on day 1 immediately prior to ifosfamide administration. For the quantification of ifosfamide and dechloroethyl metabolites in plasma analysis, specific fresh blood samples were drawn during two chemotherapy cycles, once during cycle 1 and again between cycles 3 and 5. At both cycles, one blood sample was drawn before each infusion of ifosfamide, one between 5 and 15 min after the end of infusion, and a final sample between 2 and 3 h after the completion of the infusion (for 2–3 consecutive days depending on the chemotherapy regimen, i.e. IVA or VIDE). Samples were immediately centrifugated and plasma was collected and frozen down at -80 °C.

#### Urine

24-hour urines were collected (from spontaneous urine release), starting 24 h prior to the initiation of ifosfamide, and every 24 h thereafter up to 24 h after the end of the last ifosfamide infusion. Each 24-hour urinary volume was measured. Urine samples were stored at + 4 °C and used for renal function assessment. A urine aliquot was used for NMR spectrometry. Another urine aliquot was immediately processed for quantification of ifosfamide and dechloroethylated metabolites.

### Renal function

Plasma and urine samples were collected for measurement of creatinine, cystatin C, sodium, potassium, glucose, bicarbonates, protein, calcium, phosphate, magnesium, uric acid, and osmolality. All plasma creatinine measurements were performed with traceable methods in accordance with the National Institute of Standards and Technology (isotope-dilution mass spectrometry-calibrated).

Estimated glomerular filtration rate (eGFR) was calculated using the creatinine-based bedside Schwartz formula before and after each chemotherapy cycle [11]. For the long-term renal evaluation, a real GFR measurement (mGFR) was performed by plasma iohexol clearance [12].

Fractional excretion (FE) of glucose, uric acid, and phosphate were calculated, as well as the ratio of tubular maximum phosphate reabsorption per GFR (TmP/GFR), and the calcium/creatinine, albumin/creatinine, and β2-microglobulin/creatinine urinary ratios.

Decreased GFR or increased plasma levels of creatinine, cystatin C, and albumin/creatinine ratio, indicated glomerular abnormalities. Tubular kidney disorders were characterized by the presence of one or several abnormalities: renal tubular acidosis (decreased plasma bicarbonates), renal phosphate wasting (decreased TmP/GFR), hypouricemia with increased FE of uric acid, increased FE of glucose, renal calcium wasting (increased urinary calcium/creatinine), increased β2-microglobulin/creatinine, decreased urinary concentration capacity (fasting urinary osmolality < 700 mOsm/kg).

#### Acute renal toxicity

A comprehensive evaluation of renal function was conducted on plasma and urine samples prior and following ifosfamide administration, including glomerular and renal tubular function. Acute renal toxicity was defined by a clinically significant change in glomerular and/or tubular parameters between the pretreatment and the 24-hour post-chemotherapy evaluations (i.e., relative change > 50% for one or several parameters), based on the definition of acute kidney injury proposed for changes in serum creatinine [13]. As these research data were not available throughout chemotherapy, the treatment doses were thus adapted according to the current recommendations of the therapeutic protocols but were not based on results of our research.

#### Long-term renal toxicity

After completion of their ifosfamide-based chemotherapy regimen, data pertaining to each patient’s renal function were collected as deemed necessary by the clinical team during at least 5 years. Data from the latest available renal assessment performed for each patient is presented.

Blood pressure was measured 3 times on a patient lying down and calm; the lowest systolic and diastolic results were recorded, as recommended. Arterial hypertension was defined as a systolic or diastolic blood pressure above the 95th percentile for children under 18 years, or above 140/90 mmHg for the older ones [14].

According to the Kidney Disease: Improving Global Outcomes (KDIGO) guidelines for the evaluation and management of chronic kidney disease (CKD), decreased GFR < 90 ml/min/1.73 m^2^ and increased urinary albumin/creatinine ratio > 3 mg/mmol were considered as abnormal [15].

Overall chronic nephrotoxicity was quantified using the score from Skinner et al. calculated from four parameters of the renal function: GFR; TmP/GFR; bicarbonates; early morning urine osmolality [16]. Each measure was scored from 0 to 4, with 0 representing no, 1 mild, 2–3 moderate, and 4 severe toxicity within each individual aspect of renal damage. Total score (i.e., sum of the four parameters) ranged from 0 to 16, with 0 indicating no, 1–3 mild, 4–7 moderate, and ≥ 8 severe nephrotoxicity.

### Urinary metabolomics using proton-NMR (^1^H NMR) spectroscopy

High resolution ^1^H NMR (Bruker AM-500 WB spectrometer) was used to analyze urine samples from the patients and identify several metabolites excreted in the urines [17]. Briefly 450 µl urine were mixed with 50µL of D4 trimethylsilyl propionic acid (0.29 µmole of TSP which serves as standard), and with 100 µl deuterium oxide (D_2_O). Assignments of resonances were made considering chemical shifts relative to TSP and spin-spin coupling patterns. Major metabolites were identified on ^1^H NMR spectra of urines. They were also confirmed by adding standard materials to the samples. Resonance intensities were determined on the basis of peak height measurements, taking into account the multiplicity of the signals. The resonance intensities were then corrected for the number of protons contributing to the signal, and calculated in µmole with the TSP standard. To note, among all identified metabolites, only data from betaine and glycine that are considered as potential markers of renal toxicity were presented in this study [18–21].

### Aldehyde dehydrogenase (ALDH) enzymatic activity

ALDH enzymatic activity was measured before each cycle. Peripheral blood lymphocytes were isolated from patients’ blood sample using the Uni-Sep Lymphoprep technique according to manufacturer instructions (Eurobio scientific). Aliquots of lymphocytes were frozen at -80 °C and batched measurements of ALDH enzymatic activity were performed on thawed samples [22]. A highly sensitive technique using enzymatic recycling amplification was used [23, 24]. Aliquots of the samples were used for protein determination with the bicinchoninic acid method and results were expressed in µmoles per g protein for 30 min at 37 °C.

### Quantification of ifosfamide and dechloroethylated metabolites

The plasma sample preparation was performed by protein precipitation. To 100 µL of plasma, 50 µL of a methanol solution containing cyclophosphamide [^2^H_8_] (Alsachim, Illkirch, France) was added as an internal standard. Then, 0.5 mL of mixture acetonitrile/methanol (75/25) was added, samples were vortexed for 30 s, and centrifugation was performed at 13,000×g for 5 min. The supernatant was evaporated to dryness under a nitrogen stream and the residue was reconstituted in 0.2 mL of mobile phase before injection into liquid chromatography coupled to tandem mass spectrometry (LC-MS/MS) apparatus. Analytical conditions for LC-MS/MS analysis derived from a previous published work [25]. Ifosfamide, 2-dechloro- and 3-dechloro-ethyl-ifosfamide were purchased from Niomech (IIT GmbH, Bielefeld, Germany) as pure compounds for standard calibrations. The urine sample preparation involved dilution (1:10) with water and the addition of the internal standard.

### Statistical analysis

Since this is a preliminary screening study and little or none is known about the ALDH enzymatic activity and the ^1^H-NMR profile of urines from children receiving ifosfamide-based chemotherapy regimen, data collection from up to 15 participants was considered relevant for the initial assessment of enzymatic profiles and the ^1^H-NMR metabolomics.

Renal function, ifosfamide’s metabolites, and ALDH enzymatic activity were carefully examined to identify one or several profiles of children at high risk of nephrotoxicity. Due to the nature of the study and the small number of patients, only a descriptive analysis was performed.

## Results

### Participants’ characteristics

**Fig. 1:**
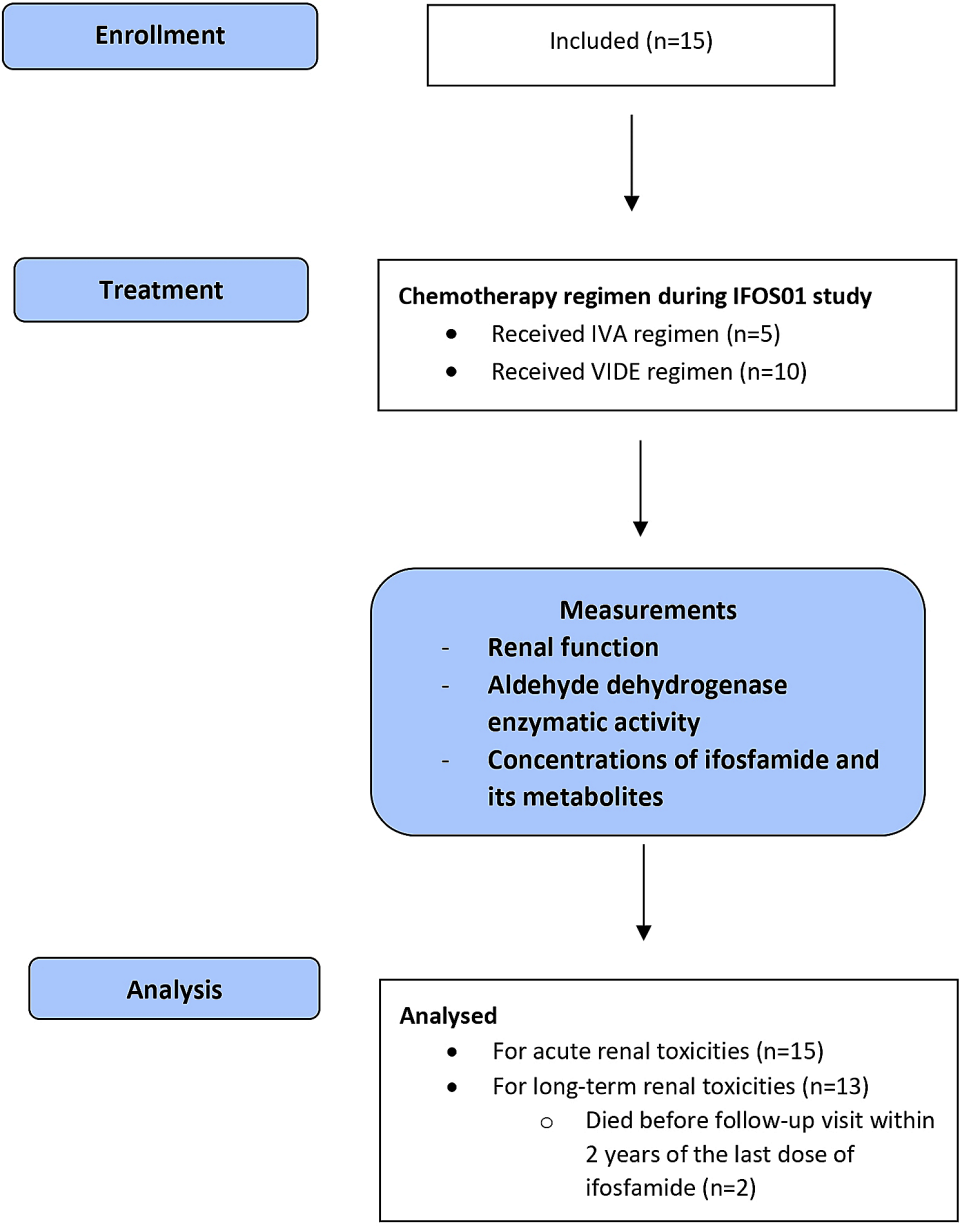
Flow diagram. IVA regimen, Ifosfamide, vincristine and actinomycin D; VIDE regimen, Etoposide, ifosfamide, adriamycine and vincristine

Between October 2009 and January 2015, 15 children were included in the study (Fig. 1). Main clinical characteristics are presented in Table 1. Median age at diagnosis was 13 years (range: 8–17) and two thirds were male. Ten children had Ewing sarcoma, 4 had embryonal rhabdomyosarcoma, and 1 had alveolar rhabdomyosarcoma. The majority (73.3%) had no metastasis at diagnosis.

**Table 1:** Participants’ demographics and clinical characteristics

Patient	Age at diagnosis (years)	Sex	Diagnosis	Metastases at diagnosis	Chemotherapy regimen	Total number of cycles with ifosfamide^a^	Cumulative dose of ifosfamide (g/m^2^)^a^	Time interval between last dose of ifosfamide and long-term evaluation of renal function (months)	Long-term nephrotoxicity^c^
**1**	17	M	Emb. Rhab.	No	IVA	7	62	64	Grade 1
**2**	13	F	Ewing sarcoma	Yes	VIDE	14	97	42	Grade 1
**3**	12	M	Ewing sarcoma	No	VIDE	7	60	89	No
**4**	12	M	Ewing sarcoma	No	VIDE	7	59	56	No
**5**	14	F	Emb. Rhab.	No	IVA	9	54	98	Grade 1
**6**	13	F	Ewing sarcoma	Yes	VIDE	11	80	Not available^b^	Not evaluable^b^
**7**	12	M	Ewing sarcoma	No	VIDE	6	54	29	No
**8**	8	M	Ewing sarcoma	Yes	VIDE	14	102	Not available^b^	Not evaluable^b^
**9**	10	M	Emb. Rhab.	No	IVA	4	24	31	Grade 1
**10**	10	M	Alv. Rhab.	No	IVA	9	54	13	Grade 1
**11**	17	M	Ewing sarcoma	No	VIDE	6	53	21	Grade 1
**12**	16	F	Ewing sarcoma	Yes	VIDE	14	99	30	Grade 1
**13**	17	M	Emb. Rhab.	No	IVA	9	50	74	Grade 2
**14**	11	M	Ewing sarcoma	No	VIDE	7	59	14	No
**15**	16	F	Ewing sarcoma	No	VIDE	7	54	21	No

Emb. Rhab., Embryonal rhabdomyosarcoma; Alv. Rhab., Alveolar rhabdomyosarcoma; IVA regimen, Ifosfamide, vincristine and actinomycin D; VIDE regimen, Etoposide, ifosfamide, adriamycine and vincristine

^a^Including cycles and doses of ifosfamide received during IFOS01 study (corresponding to induction treatment) and after induction. ^b^Patient’s death before long-term follow-up visit. ^c^Nephrotoxicity using the score from Skinner et al.

The total cumulative dose of ifosfamide received by each child throughout treatment (i.e. including doses during induction treatment and supplementary doses) is presented in Table 1. Nine children (60%) received additional doses of ifosfamide following induction regimen: 8 patients with Ewing sarcoma received ifosfamide-based consolidation therapy after surgery and 1 patient with rhabdomyosarcoma (n°1) who had insufficient response to IVA benefited from a salvage chemotherapy with ifosfamide. In total, the cumulative ifosfamide dose ranged from 24 to 102 g/m^2^ (median 59), given over 4 to 14 cycles (median 7).

To note, one child (n°13) had a dose reduction secondary to renal toxicity as per therapeutic protocol at cycle 9. Three children (20%) died because of tumoral progression within 2 years of the last ifosfamide administration, (patients n°6, n°8 and n°10 at 12, 18 and 20 months from the last dose, respectively). Two of these 3 children did not have a long-term assessment of renal function after chemotherapy completion.

### Renal function

#### Acute toxicity

Median values of both glomerular and tubular parameters, before and 24 h after cycles, are presented in Table 2.

**Table 2:** Renal function parameters before and after administration of ifosfamide and acute changes during chemotherapy cycles

	Pre-chemotherapy		24 h-post-chemotherapy		Acute change* (%)
	n	Median (min-max)	n	Median (min-max)	Median (min-max)
**Glomerular parameters**					
eGFR (ml/min/1.73m^2^)	89	126 (54–171)	81	136 (81–206)	8 (-25-66)
Plasma creatinine (µmol/L)	89	45 (29–113)	81	42 (28–75)	-7.3 (-39.7-33.3)
Plasma cystatin C (mg/L)	68	0.7 (0.5–1.1)	61	0.8 (0.3–1.1)	5.5 (-23.0-34.8)
Urine albumin/creatinine (mg/mmol)	90	1.2 (0.1–8.4)	89	0.1 (0.1–18.3)	0.0 (-99.4-15008.7)
**Tubular parameters**					
Plasma sodium (mmol/l)	89	139 (134–145)	81	137 (132–143)	-1.4 (-6.3-1.5)
Plasma potassium (mmol/l)	89	3.8 (3.1–4.7)	81	3.7 (2.9–6.9)	-2.4 (-38.3-76.9)
Plasma bicarbonate (mmol/l)	89	25.5 (18.1–30.3)	81	24.5 (19.4–30.3)	-2.7 (-27.7-28.2)
Plasma protein (mg/l)	89	70 (60–83)	81	69 (60–82)	0.0 (-13.3-13.0)
Plasma calcium (mmol/l)	89	2.3 (2.1–2.6)	81	2.3 (2.0-2.6)	0.4 (-9.2-12.6)
Plasma magnesium (mmol/l)	89	0.8 (0.6-1.0)	81	0.8 (0.6–1.1)	1.3 (-19.2-21.3)
Plasma phosphate (mmol/l)	89	1.4 (0.8–1.7)	81	1.1 (0.7–1.5)	-13.7 (-48.3-32.3)
Fractional excretion of phosphate (%)	89	9.5 (3.6–19.1)	81	18.8 (8.3–37.0)	120.9 (-20.7-351.3)
Tmp/GFR (mmol/l)	89	1.2 (0.6–1.6)	81	0.9 (0.4–1.3)	-22.5 (-62.7-18.1)
Plasma uric acid (µmol/L)	89	184 (105–410)	81	136 (72–287)	-27.7 (-56.1-4.5)
Fractional excretion of uric acid (%)	89	8.6 (2.7–19.0)	81	24.8 (9.3–41.7)	193.0 (12.7-513.8)
Fractional excretion of glucose (%)	89	0.1 (0.0-0.4)	86	0.1 (0.0–23.0)	75.4 (-87.1-229843.1)
Urine calcium/creatinine (mmol/mmol)	90	0.5 (0.1–1.9)	88	0.9 (0.1–2.1)	70.3 (-56.4-858.2)
Urine β2-microglobulin/creatinine (mg/mol)	90	6.0 (4.6-115.3)	89	417.2 (6.0-2743.8)	4704.8 (-74.6-45630.7)
Urine glycine/creatinine	63	0.2 (0.0-2.1)	61	0.9 (0.0-4.6)	293.1 (-74.5-3225.6)
Urine betaine/creatinine	62	0.0 (0.0-0.5)	62	0.3 (0.0-1.1)	802.7 (-59.3-5991.0)

*Relative change between pre-chemotherapy (i.e., value before ifosfamide infusion) and 24 h after completion of chemotherapy cycle

*eGFR*, Estimated glomerular filtration rate (Schwartz 2009); *TmP/GFR*, Renal tubular maximum reabsorption rate of phosphate

After each cycle, plasma level of creatinine decreased and eGFR increased, probably linked to hyperhydratation. Plasma cystatin C slightly increased (median: +5.5%), and the urine albumin/creatinine ratio was unchanged, indicating no clinical abnormality of glomerular function.

All children presented signs of tubular toxicity in several cycles. In particular, there was consistent decrease in phosphate and uric acid levels in blood along with major increase in FE of phosphate and uric acid, and a moderate decrease in Tmp/GFR (median: -22.5%). FE of glucose and β2-microglobulin/creatinine increased in a clinically significant manner after chemotherapy.

In addition, urinary excretion of the amino acid glycine and betaine was measured using NMR, allowing to calculate the glycine/creatinine and betaine/creatinine ratios. Both ratios dramatically increased after chemotherapy cycles, strengthening impairment of tubular function (Table 2).

Overall, acute toxicities were similar throughout cycles and reversible (i.e., with a return to baseline level at the start of the next cycle), except for one patient with persistent impaired glomerular function (n°13).

#### Long-term toxicity

Long-term evaluation of renal function was performed at a median follow-up of 31 months (range: 13–98) after last administration of ifosfamide. Median values of renal function parameters from last evaluation are given in Table 3.

**Table 3:** Long-term renal follow-up after completion of treatment with ifosfamide

	n	Median (min-max)	Number of abnormal values (%)
**General parameters**			
Systolic BP percentile (%, N < 95th percentile)	13	62 (1–93)	0
Diastolic BP percentile (%, N < 95th percentile)	13	78 (10–95)	0
**Glomerular parameters**			
mGFR (ml/min/1.73m^2^, N > 90)	13	87 (55–111)	7 (54%)
Plasma creatinine (µmol/L, N depending of age and sex)	13	69 (45–102)	0
Urine albumin/creatinine (mg/mmol, N < 3)	11	1.4 (0.6–12.0)	3 (27%)
**Tubular parameters**			
Plasma sodium (mmol/l, N 136–145)	13	139 (137–146)	0
Plasma potassium (mmol/l, N 3.5–4.8)	12	4.0 (3.6–4.5)	0
Plasma bicarbonate (mmol/l, N 22–29)	12	24.2 (21.0–30.0)	0
Plasma calcium (mmol/l, N 2.10–2.55)	13	2.4 (2.2–2.7)	0
Plasma magnesium (mmol/l, N 0.70–0.91)	13	0.9 (0.8–86.0)	0
Plasma phosphate (mmol/l, N depending of age)	12	1.2 (0.9–1.9)	0
Fractional excretion of phosphate (%, N < 20)	12	10.2 (2.7–25.9)	1 (8%)
TmP/GFR (mmol/l, N > 0.8 mmol/L)	12	1.1 (0.8–1.5)	0
Plasma uric acid (µmol/L, N depending of age and sex)	13	213 (129–333)	4 (31%)
Fractional excretion of uric acid (%, N < 11.26)	13	8.6 (3.2–16.0)	3 (23%)
Fractional excretion of glucose (%, N < 0.1)	11	0.1 (0-0.2)	1 (9%)
Urine calcium/creatinine (mmol/mmol, N < 0.7)	13	0.3 (0.1–0.7)	0
Urine β2-microglobulin/creatinine (mg/mol, N < 40.7)	9	36.0 (2.0-299.0)	4 (44%)
Urine osmolality (mOsm/kg, N > 700)	12	872.5 (591.0-1024.0)	1 (8%)

*BP*, Blood pressure; *mGFR*, Real glomerular filtration rate; *TmP/GFR*, Renal tubular maximum reabsorption rate of phosphate. N indicates normal value or range for each parameter

The most common abnormality was reduced mGFR observed in 7 out of 13 evaluable patients (54%). Three patients had microalbuminuria, indicating glomerular dysfunction. Two patients showed increased FE of uric acid in the presence of hypouricemia. Four of the 9 patients (44%) with evaluable β2-microglobulin/creatinine had abnormally increased level of this ratio. No patient had abnormal plasma phosphate level nor low renal tubular threshold for phosphate (Tmp/GFR).

Of the 13 evaluable patients for overall chronic nephrotoxicity grading, 8 (62%) showed mild nephrotoxicity using the definition from Skinner et al. [16]. Six patients had grade 1 toxicity for GFR and another one developed a toxicity of grade 2 for GFR. One patient had distal renal tubular disorder (i.e. urine osmolality ≤ 700 mOsm/kg). No patient showed impaired Tmp/GFR nor renal tubular acidosis.

To note, all but three children had a single long-term assessment of renal function after ifosfamide completion. For three children (n°3, 5 and 13), another measurement had been carried out earlier (data not shown), indicating renal damage that persisted over time for two children (n°5 and 13) and no toxicity on both evaluations for one child (n°3).

### ALDH enzymatic activity in the peripheral blood lymphocytes

**Fig. 2:**
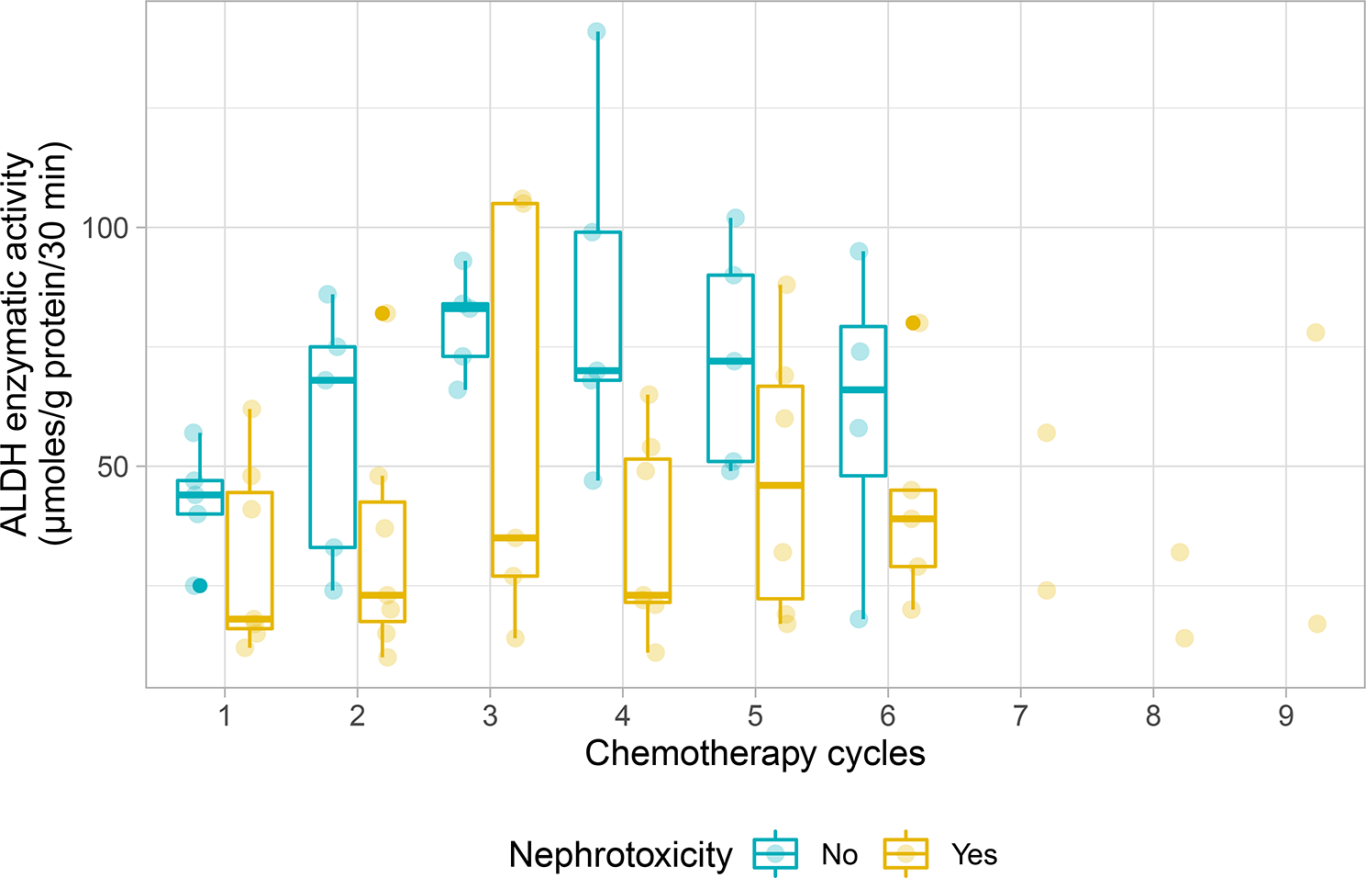
Aldehyde dehydrogenase (ALDH) enzymatic activity before each chemotherapy cycle, according to overall chronic nephrotoxicity (nephrotoxicity status was determined using the definition from Skinner et al. [16])

The individual change in ALDH enzymatic activity from baseline to subsequent cycles did not permit the identification of specific profiles, owing to both inter- and intra-individual variations in ALDH. However, considering the long-term renal status using the definition from Skinner et al. [16], the distribution of values in Fig. 2 showed generally lower ALDH enzymatic activity in children with chronic nephrotoxicity than in those without chronic nephrotoxicity across cycles, although no formal comparison of repeated measures was performed.

### Concentrations of ifosfamide and its metabolites

**Fig. 3:**
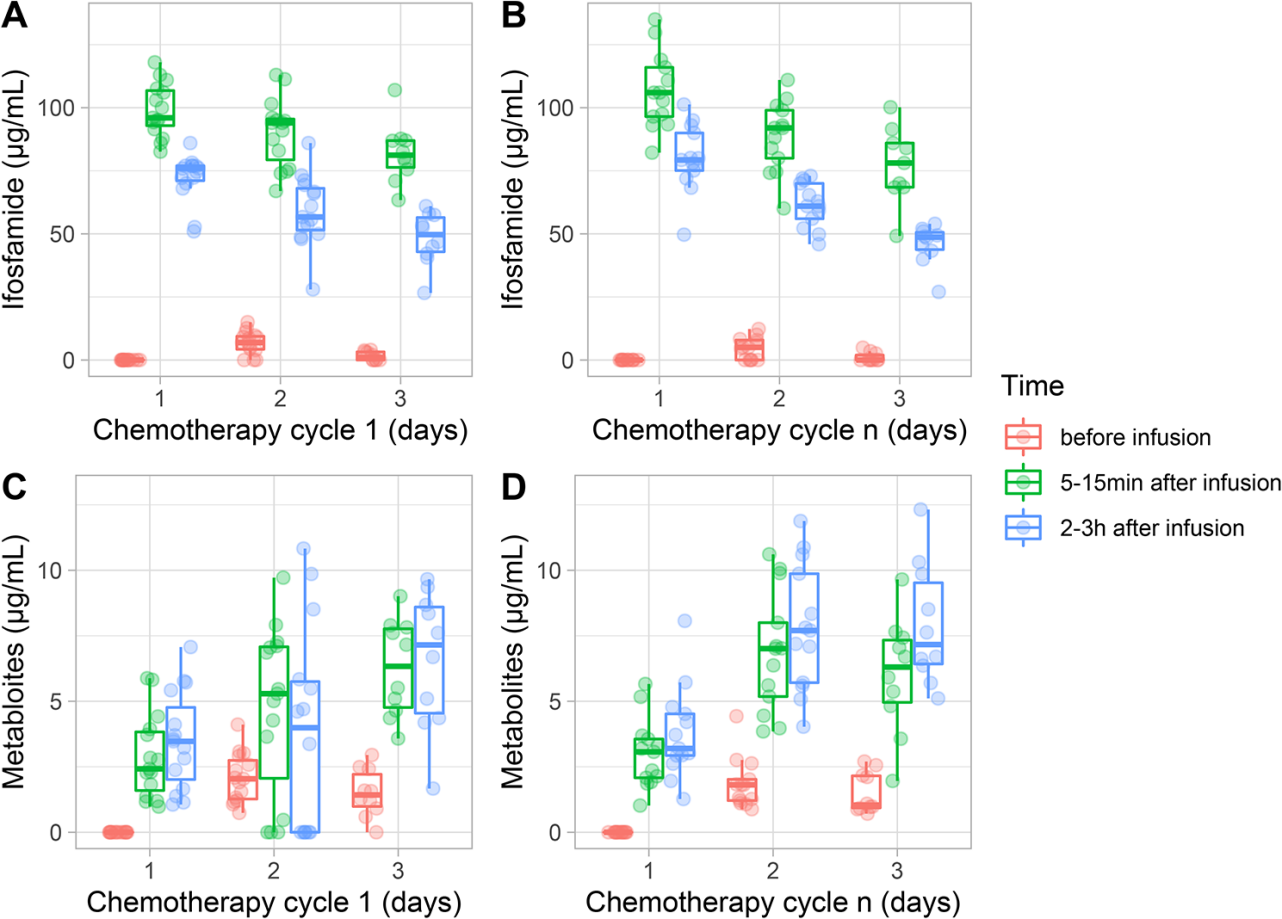
Plasma concentration of ifosfamide (**A**, **B**) and of 2- and 3-dechloroethyl ifosfamide metabolites (**C**, **D**) at cycle 1 and cycle n. The figures depict measures before infusion of ifosfamide, between 5–15 min and between 2-3 h after infusion of ifosfamide. *N.B.: only children with VIDE regimen had measurements at D3*

Concentrations of ifosfamide and its dechloroethylated metabolites in blood at cycle 1 and cycle n (i.e. cycle 3 to 5 depending on patient) are plotted in Fig. 3. Over a 2- to 3-day cycle, concentration of ifosfamide decreased and concentration of the 2- and 3-dechloroethylated metabolites increased. In urine, all patients had similar concentration profiles for both ifosfamide and its metabolites in early and late chemotherapy cycles (see Additional file 1). There were approximately twice as many 3-dechloroethyl as 2-dechloroethyl metabolites in blood and urine. Intact ifosfamide was found predominantly over the 2- and 3-dechloroethylated metabolites in urine.

## Discussion

This exploratory study sought early urinary markers of chronic renal toxicity secondary to ifosfamide-based chemotherapy, and explored the potential role of ALDH in ifosfamide-induced nephrotoxicity.

In our cohort, all children experienced acute renal toxicity during cycles, mostly of the proximal tubule, with a return to baseline level at the start of the next cycle. After a median follow-up of 3 years, more than half of the children also presented long-term renal toxicities affecting glomerular and tubular functions. In particular, 8 out of 13 children of the cohort developed mild nephrotoxicity using the definition from Skinner et al. [16], among which 7 showed decreased glomerular filtration rate. Moreover, 7 of 13 children presented biological signs of tubular dysfunction without clinical symptoms and observed only with specific tubular assessment. Thus, our study suggested that some children treated with ifosfamide had a persistent renal damage after chemotherapy completion, with a marked decrease in glomerular function. These findings are consistent with those of previous studies on similar cohorts [10, 26–29].

Similarly to our study, a 10-year follow-up study from Skinner et al. suggested that clinically significant tubular toxicity resolved by ten years after ifosfamide completion, while glomerular function assessed through measured GFR deteriorated in some patients [29]. In another study from Skinner et al. that explored renal function of 11 children with cancer either during the course of ifosfamide (*n* = 6) or 2 to 15 months after completion of treatment (*n* = 5), 6 of 11 children had abnormal glomerular filtration rates and all 11 developed tubular damage [26]. Rossi et al. carefully monitored renal function of 75 patients during a median follow-up of 31 months after completion of ifosfamide treatment, showing that all 5 patients who developed Fanconi syndrome presented subclinical forms of tubulopathy in advance [28]. In fact, a Fanconi syndrome appeared within the first 3 years following the end of treatment, while subclinical tubulopathy developed within the first 2 years after treatment completion.

Several studies described acute and late renal toxicities related to ifosfamide use in children. However, due to methodological limitations, the available evidence does not allow precise determination of the prevalence of early and long-term renal damage after treatment [3, 30]. Moreover, only a few studies assessed early markers of renal dysfunction that might predict the development of late nephrotoxicity [31]. To date, a decrease in amino acid reabsorption is the most common ifosfamide-induced tubular dysfunction, and early hyperaminoaciduria could thus represent a sensitive parameter of chronic tubulopathy [28, 32]. Hence, we proposed to use NMR for the early detection of subtle, ifosfamide-induced tubular abnormalities including hyperaminoaciduria [33, 34].

Proton NMR spectroscopy allowed for the assessment of the urinary metabolome. Only two metabolites were described, as they appeared relevant to detect risk of progressive chronic kidney disease. The first metabolite, betaine, is a renal osmolyte from the distal renal tubular cells that serves to counteract the osmotic forces of urine. Increased urinary excretion of betaine was previously reported in patients with renal disease [18]. The second one was the amino acid glycine, which is reabsorbed in the proximal tubule and should not be excreted in the urine or only in minimal quantities. Its presence could thus reflect proximal tubular injury, as it has been shown in cisplatin-treated rats [19, 20] but also in ifosfamide-treated adult sarcoma patients [21]. Urinary excretion of both betaine and glycine significantly increased after chemotherapy in the same way as the other conventional makers of tubular damage, strengthening potential renal tubular dysfunction secondary to ifosfamide.

It was postulated that the nephrotoxic effects of ifosfamide may result from increased exposure of the renal cells to its toxic metabolite chloroacetaldehyde, which could be related to a decrease in the activity of ALDH. ALDH could indeed play a major role in the detoxification pathway of chloroacetaldehyde. The main site for ifosfamide detoxification is the liver where ALDH is the primary enzyme contributing to the transformation of acetaldehyde (nephrotoxic) in acetate (nontoxic) [35]. Inhibition of ALDH was shown to induce a significant increase in cytotoxicity in chloroacetaldehyde-treated rat hepatocytes [36]. Moreover, it seems that lymphocytes are a reliable indicator of an individual’s ALDH phenotype, and the leukocyte ALDH activity might be similar to the human liver mitochondrial isozyme (ALDH I) [37, 38]. Hence, ALDH enzymatic activity was measured in the peripheral blood lymphocytes as a measure of the body’s detoxification capabilities towards ifosfamide-derived chloroacetaldehyde.

The analysis of ALDH enzymatic activity during chemotherapy revealed high inter- and intra-individual variations across cycles. However, patients with lower ALDH activity during treatment showed greater degree of overall chronic nephrotoxicity, which might indicate a poor capacity of chloroacetaldehyde detoxification due to low ALDH activity in patients who developed chronic nephrotoxicity. This suggests a possible role of ALDH in renal toxicity that could be explored in another population. In addition, evaluation of ALDH polymorphism as a potential individual predictor of ifosfamide’s toxicity could permit to investigate the genetic basis for differential metabolism between patients receiving ifosfamide.

An increase in chloroacetaldehyde levels might also result from an increased production by liver and kidney. Ifosfamide and 2- and 3-dechloroethyl-ifosfamide were measured as indicators of chloroacetaldehyde production. Numerous studies have reported pharmacokinetic data following administration of ifosfamide in patients with cancer, including children [39–42]. In all studies, ifosfamide was subject to autoinduction, which led to increased clearance over time. In our cohort, plasma concentration of ifosfamide decreased while its dechloroethylated metabolites increased during both early and late chemotherapy cycles. The later observation supports an enzymatic induction of ifosfamide that could potentially lead to an increased production of toxic chloroacetaldehyde. However, all patients had similar concentration profiles for ifosfamide and metabolites, which did not allow discriminating between patients who were at increased risk of chronic nephrotoxicity and those who were not.

Ifosfamide remains a major cancer drug widely used for the treatment of pediatric solid tumors. No drug has been approved to prevent or treat ifosfamide-induced nephrotoxicity. Recommendations for the long-term surveillance of renal function have been established for patients treated with ifosfamide-based regimen [43], but the prevention of nephrotoxicity is still a challenge. To note, tubular reabsorption of phosphate (or fractional excretion of phosphate) is traditionally used to assess tubular function in clinical practice but it depends on age and diet. The TmP/GFR, corresponding to the renal phosphate threshold, should be preferred to evaluate tubular phosphate handling as it is independent of dietary phosphate intake [44]. Measurement of urinary excretion of amino acid would be a more sensitive indicator of tubular dysfunction but is cumbersome and not possible in daily practice. Moreover, the ability to predict the risk of renal toxicity in a specific patient remains elusive. The identification of early markers of renal toxicity along with the recognition of a genetic background (i.e., enzymatic polymorphism) predisposing patients to suboptimal ifosfamide metabolism should unquestionably help clinicians to alter and eventually reverse the course of ifosfamide-induced nephrotoxicity [13].

The strengths of this study include precise and valuable information on ALDH enzymatic activity in children receiving ifosfamide-based chemotherapy. To the best of our knowledge, no study investigated the expression of ALDH enzymatic activity in blood samples of patients treated with ifosfamide and its possible link with long-term nephrotoxicity. Moreover, renal function was fully assessed during chemotherapy cycles and long after the end of chemotherapy. Hence, overall chronic nephrotoxicity could be graded using the definition from Skinner et al., allowing to calculate a standardized score reflecting the severity of clinically relevant ifosfamide nephrotoxicity [45].

Our study also had some limits that should be acknowledged. In particular, the very small sample size of the cohort limits the interpretation of the findings regarding the potential implication of ALDH in long-term nephrotoxicity. The small sample also prevented to establish a link between the severity of acute renal toxicities and the development of late nephrotoxicity. The age limit was set at 6 years so that daytime and nighttime continence was acquired for 24-hour urine collection, but it would also have been interesting to explore the renal function of younger patients including infants [46]. The 5-year inclusion period highlights the complexity of recruiting young patients with solid tumors and set up clinical studies in these indications. Furthermore, only the 2- and 3-dechloroethyl-ifosfamide were measured as indicators of chloroacetaldehyde production, while other unmeasured ifosfamide-derived metabolites could be potentially toxic for the kidney.

## Conclusions

NMR did not offer clear advantage over conventional methods for early detection of renal dysfunction, and quantification in blood of ifosfamide and its main metabolites failed to identify at-risk patient profiles. Although no conclusion can be drawn on the role of ALDH in long-term nephrotoxicity, the observed results encourage further exploration of the enzymatic activity of ALDH as well as its polymorphism in larger cohorts.

### Electronic supplementary material

Below is the link to the electronic supplementary material.

Supplementary Material 1

## Data Availability

Data generated during the current study are available from the corresponding author on reasonable request.

## Abbreviations

ALDH: Aldehyde dehydrogenase
FE: Fractional excretion
GFR: Glomerular filtration rate
IVA: Ifosfamide, vincristine and actinomycin D
NMR: Nuclear magnetic resonance
TmP/GFR: Ratio of tubular maximum phosphate reabsorption per glomerular filtration rate
TSP: Trimethylsilyl propionic acid
VIDE: Vincristine, ifosfamide, adriamycine and etoposide
