# Supplementary material for: Renal toxicity of ifosfamide in children with cancer: an exploratory study integrating aldehyde dehydrogenase enzymatic activity data and a wide-array urinary metabolomics approach
Source: BMC Pediatr. 2024 Mar 19;24:196. doi: 10.1186/s12887-024-04633-1 (PMC10949630; doi:10.1186/s12887-024-04633-1)
Supplement: Supplementary file 1 — Supplementary Material 1 [file 12887_2024_4633_MOESM1_ESM.docx]

**Additional file 1.** Ratio of 2- and 3-dechloroethyl ifosfamide metabolites / ifosfamide in urine at cycle 1 and cycle n.

| **Patient** | **Cycle 1** | **Cycle n** |
| --- | --- | --- |
| 1 | 0.69 | Not available |
| 2 | 0.75 | Not available |
| 3 | 0.51 | 0.47 |
| 4 | 0.62 | 0.41 |
| 5 | 0.61 | 0.45 |
| 6 | Not available | Not available |
| 7 | 0.69 | 0.54 |
| 8 | 0.72 | 0.59 |
| 9 | 0.66 | 0.82 |
| 10 | 0.83 | 0.77 |
| 11 | 0.70 | 0.66 |
| 12 | 0.64 | 0.63 |
| 13 | 0.76 | Not available |
| 14 | 0.83 | 0.47 |
| 15 | 0.61 | 0.52 |
